# Does the socio-demographic profile of patients limit access to bariatric surgery?

**DOI:** 10.1007/s40519-021-01285-3

**Published:** 2021-08-24

**Authors:** Viviane Richard, Christof Stähli, Guillaume Giudicelli, Marc Daniel Worreth, Nicole Krähenbühl, Emilie Greiner, Chrysoula Papastathi, Michele Diana, Alend Saadi

**Affiliations:** 1 Surgery Department, Neuchâtel Hospital, Neuchâtel, Switzerland; 2 Obesity and Metabolic Diseases Centre, Neuchâtel Hospital, Neuchâtel, Switzerland; 3 IRCAD, Research Institute Against Digestive Cancer, Strasbourg, France; 4 Surgery Department, Strasbourg University Hospital, Strasbourg, France; 5 Faculty of Biology and Medicine, University of Lausanne, Lausanne, Switzerland; 6 Service of Populational Epidemiology, Geneva University Hospital, Geneva, Switzerland; 7 Visceral Surgery Service, Geneva University Hospital, Geneva, Switzerland

**Keywords:** Bariatric surgery, Non-operation, Socio-demographic clusters

## Abstract

**Purpose:**

Surgery remains the only treatment allowing for a significant and sustainable weight loss in case of severe obesity. Patients undergo a specific multidisciplinary preparation and selection before the operation. This study aims to correlate the psychosocial profile with the likelihood of undergoing bariatric surgery in patients enrolled in the preparation program of a Swiss reference center.

**Methods:**

All patients referred to an obesity center between January 1, 2016, and June 30, 2017, seeking a first bariatric procedure were included. Socio-demographic data, BMI, preoperative psychological and dietary evaluations were collected. Usually, the preoperative process lasts 1 year. Patients who left the preparation or who had not undergone surgery after more than 2 years of follow-up were considered withdrawers. Surgery completion predictors were reviewed with bivariate analysis and socio-demographic clusters established using the K-means method.

**Results:**

Out of a total of 221 patients, 99 (45%) patients had not undergone bariatric surgery 2 years after their first consultation. The patients were divided into four distinct socio-demographic clusters, among which a particularly deprived one. Criteria such as unfavorable psychological (*p* < 0.001) and dietary (*p* < 0.001) evaluations, and male gender (*p* < 0.05) were significantly associated with non-operation, unlike socio-demographic indicators and clusters (*p* > 0.1).

**Conclusion:**

Almost half of the patients starting a bariatric program are not operated on, which is related to an unfavorable psychological or dietary evaluation and to the male gender. This study also demonstrates that a significant share of patients combines several factors of social deprivation, without influencing the likelihood of surgery completion.

**Level of evidence:**

Level V: Descriptive study.

## Introduction

Severe obesity is a metabolic disease and a well-known risk factor for multiple other chronic disorders [1]. It also presents an important societal dimension, as it is more common among deprived socio-economic subgroups of the population and constitutes a factor of social exclusion in developed countries [2].

Bariatric surgery remains the only effective and long-term treatment of severe obesity [3–7]. However, obesity surgery is still disregarded by many, including healthcare professionals [8]. Patients must overcome an important psychological stigma before deciding to consult a bariatric center, even though weight loss surgery is highly regulated. In Switzerland, among different criteria, we can mention a BMI ≽ 35 kg/m^2^ with comorbidities and an unsuccessful non-operative treatment [9]. An adequate eating behavior and psychological stability are required for several months before surgery, imperatively supported by a multidisciplinary team approach. Nevertheless, numerous initially motivated patients do not achieve the preoperative process and do not benefit from the bariatric treatment. In weight loss intervention programs, the most common attrition reasons are practical difficulties, such as family or work problems, as well as living far from the center [10]. The socio-demographic profile of patients could be associated with such difficulties.

Socio-demographic characteristics of people with obesity have already been investigated in the general population [2, 11–13]. However, we do not clearly know how these characteristics are reflected in patients referred to a bariatric center for surgery. The presence of patterns in their distribution among patients and their possible influence on patients’ trajectories in the bariatric project remains largely unknown. Furthermore, there is limited understanding of the determinants of preoperative attrition for bariatric surgery. Existing studies often consider medical and surgical treatments altogether and do not allow to identify specific determinants of surgical attrition. Additionally, socio-demographic factors are examined separately [14–16], although several characteristics may frequently be combined and associated with a specific outcome, as found in close fields [17, 18].

Better understanding attrition may help to improve access to surgery, but also cost-effectiveness by reducing the number of potentially useless preoperative medical interventions [14]. For example, in the studied center at least 20 consultations and 8 group sessions are routinely performed prior to surgery, resulting in a substantial cost which may not be fully beneficial if surgery is not performed. As reported by Bairdain and Samnaliev [19], the cost of the preoperative follow-up may be as high as 2081 USD. In a system with universal health insurance as the Swiss one, the cost is not paid directly by patients but remains borne by society.

The aim of the present study is to identify the profile of patients applying to a Swiss referral center for bariatric surgery, as well as factors contributing to attrition.

## Methods

The institutional review board approved this retrospective cross-sectional study, and a signed informed consent was obtained from all patients.

The Obesity and Metabolic Diseases Center (OC) is part of a Swiss regional non-university teaching hospital in a peripheral setting. It offers a standard multidisciplinary preoperative and postoperative program for bariatric surgery candidates. After the initial introduction in the bariatric program by a surgeon and a dietitian, there are 8 theoretical and practical collective courses given by an endocrinologist, a dietitian, a psychologist, and a physiotherapist over approximately 6 months.

The evaluation process is then performed by the same team, consisting of at least three individual consultations with the dietitian and three consultations with the psychologist, consisting of qualitative and semi-directive clinical interviews. In addition to the pre-existing conditions, all aspects discussed in the courses are reviewed with the patients. The psychological interview examines 4 aspects of the patients’ history: cognitive, psychosocial, psychiatric, and weight evolution. The following surgery eligibility criteria are investigated: no current substance use, emotional stability, eating disorder management, understanding of the surgical procedure, as well as its potential consequences, and realistic weight loss expectation. Dietary evaluation focuses on the stability of the present eating disorder, weight stability during the process, a balanced relationship with food, and the implementation of behavior modifications towards the postoperative phase.

The operative indication and/or additional management recommendation is separately reported on a four-level scale by each the psychologist and the dietician:

- Favorable to surgery: there is no contra-indication.
- Favorable with close postoperative follow-up: there are no formal contra-indications but there is some fragility concerning dietary, psychological aspects or the patient's environment.
- Favorable with close preoperative follow-up: there is no formal contra-indication, but the dietary and psychological aspects have not been stable for long enough. A longer preparation is needed.
- Unfavorable at this stage: there is a formal contra-indication, for example, substance abuse, persistent eating disorders, unstable psychological situation or unwillingness to change lifestyle.

Based on each patient’s course, operative decision-making is finally discussed collectively throughout an “obesity board meeting”. Some patients with operative contraindications withdraw before this stage because they feel that they do not fulfill the surgery criteria. Therefore, it is delicate to distinguish if non-operation is due to self-withdrawal or medical contraindications. The preoperative program lasts about one year. Significantly longer preparations often reflect the need or wish for external follow-up, without definitive withdrawal from the OC.

All consecutive patients referred for primary bariatric surgery at the OC were retrospectively included from January 1, 2016, to June 30, 2017. Socio-demographic data such as age, gender, marital status, parenthood, country of birth, education level, and employment status, as well as BMI, preoperative psychological and dietary evaluations were manually extracted from patients’ medical records in June 2019, with a hindsight of at least 2 years or until the patient’s operation or dropout. For the present study, patients who had left the program or who had not undergone surgery after more than 2 years of follow-up were considered temporary or definitive dropouts for surgery. As non-operation reason may be complex and multifactorial, no distinction was made in this study between self-withdrawal and contraindications. Due to the Swiss federal healthcare system and to the lack of a close alternative to the OC, it is unlikely that any of the patients got operated on in another center.

To group patients with similar socio-demographic characteristics and BMI, the K-means clustering method was performed. It is an unsupervised approach to group data in a specified number of clusters K, by minimizing the within-cluster variation. A cluster is randomly assigned to each observation and the centroid of each of these clusters is computed. Each observation is then reassigned to the closest centroid, using the Euclidian distance. These steps are iterated until the within-cluster variation is minimized. To avoid a suboptimal local optimum, the complete process was repeated 40 times with different start random assignment and the result with the smallest within-cluster variation was kept.

Differences between clusters and variables related to non-operation were identified using the chi-square tests, with a significance level set at *p* < 0.05. Should there be less than five observations in a category, a Fisher’s exact chi-squared test was computed instead.

All analyses were performed with R-3.6.3.

## Results

### Patients’ socio-demographic profiles and clusters

A total of 221 patients were included in the study. Mean age was 42 (range: 17–75) and 179 (81%) were female. Mean BMI was 41.8 (range: 32–67). The most frequently reported birth countries were Switzerland (54.4%) and Portugal (21.3%). Most patients were married (47.1%) or divorced/separated (22.2%) and had children (78.4%). 31.5% attended mandatory school only, 58% had an upper secondary education level and 10.5% had tertiary level. Altogether, 52.9% were professionally active (Table 1).

**Table 1:** Descriptive and clustering analysis of patients in terms of socio-demographic and follow-up characteristics

		All N = 221	Proportion	Cluster 1^1^ N = 42	Cluster 2^1^ N = 29	Cluster 3^1^ N = 79		Cluster 4^1^ N = 71		Fisher’s exact test
		*N*	%	%	%	%		%		*P* value
Age										
17–24		16/221	7.2	23.8	0.0	1.3		7.0		0.000
25—34		48/221	21.7	54.8	10.3	15.2		14.1		
35—44		57/221	25.8	7.1	24.1	36.7		25.4		
45—54		68/221	30.8	7.1	51.7	36.7		29.6		
55 +		32/221	14.5	7.1	13.8	10.1		23.9		
Gender										
Female		179/221	81.0	69.0	0.0	100.0		100.0		0.000
Male		42/221	19.0	31.0	100.0	0.0		0.0		
Marital status										
Single		62/221	28.1	90.5	6.9	12.7		16.9		0.000
Married		104/221	47.1	4.8	69.0	60.8		47.9		
Divorced/separated		49/221	22.2	4.8	20.7	22.8		32.4		
Widowed		6/221	2.7	0.0	3.4	3.8		2.8		
Parenthood										
Yes		149/190	78.4	2.9	90.0	93.3		98.4		0.000
No		41/190	21.6	97.1	10.0	6.7		1.6		
Country of birth										
Switzerland		92/169	54.4	63.3	52.4	63.5		40.0		0.034
Portugal		36/169	21.3	10.0	14.3	23.8		27.3		
Other (21 countries)		41/169	24.3	26.7	33.3	12.7		32.7		
Education level										
Mandatory		45/143	31.5	11.1	25.0	25.9		54.8		0.007
Upper secondary		83/143	58.0	74.1	62.5	63.8		38.1		
Tertiary		15/143	10.5	14.8	12.5	10.3		7.1		
Employment status										
Employed		117/221	52.9	52.4	65.5	94.9		1.4		0.000
Stay at home		31/221	14.0	0.0	0.0	1.3		42.3		
Disability insurance		24/221	10.9	9.5	10.3	1.3		22.5		
Social aid		19/221	8.6	2.4	10.3	0.0		21.1		
Employment insurance		16/221	7.2	14.3	10.3	2.5		7.0		
At school		7/221	3.2	16.7	0.0	0.0		0.0		
Retired		7/221	3.2	4.8	3.4	0.0		5.6		
BMI (kg/m^2^)										
30—34		7/220	3.2	2.4	6.9	2.5		2.9		0.088
35—39		75/220	34.1	21.4	31.0	34.2		42.9		
40—44		82/220	37.3	38.1	27.6	48.1		28.6		
45—49		40/220	18.2	28.6	24.1	11.4		17.1		
50 +		16/220	7.3	9.5	10.3	3.8		8.6		
Psychological evaluation										
Favorable										0.806
Close postop follow-up		20/139	14.4	17.9	0.0	17.5		12.2		
Close preop follow-up		37/139	26.6	25.0	23.1	24.6		31.7		
Unfavorable		59/139	42.4	35.7	61.5	40.4		43.9		
Dietary evaluation										
Favorable										0.326
Close postop follow-up		17/99	17.2	15.8	8.3	12.5		25.0		
Close preop follow-up		31/99	31.3	31.6	16.7	37.5		30.6		
Unfavorable		41/99	41.4	42.1	75.0	31.3		38.9		

^a^Defined with socio-demographic variables and BMI (upper lines, without psychological and dietary evaluations)

Using the above-mentioned variables, different numbers of clusters were tested from 3 to 6. There were no differences between these classifications towards the operation status. Given the clinical relevance of the results and their readability, the analysis of the 4 clusters was kept.

Patients’ distribution into four distinct clusters based on socio-demographic variables (*p* < 0.05, except for BMI) is presented in Table 1. The clustering approach allowed to identify patients’ subgroups with various socio-demographic profiles. Cluster 1 was mainly composed of young (mean age = 31.4 years old), single (90.5%) people without children (97.1%), who were born in Switzerland (63.3%). An important proportion had an upper secondary education (74.1%) and all students fell in this cluster. Clusters 2 and 3, respectively, regrouped men and women with similar profiles: middle-aged married parents who were professionally active. Finally, cluster 4 was made of women with children. Compared to the rest of the patients, they tended to be more frequently born outside Switzerland (75.7%), divorced, or separated (32.4%) and with a mandatory education level (54.8%). Most of them were unemployed, 42.3% being housewives and 50.6% living on social insurances (disability, employment insurance or social aid). Psychological and dietary preoperative evaluations were not associated with clusters (p > 0.1; Table 1). A summary of the patients’ typical profile is presented for each cluster in Table 2.

**Table 2:** Main characteristics of the four clusters defined by means of socio-demographic variables and BMI

Cluster	Patients number (%)	Characteristics	
		Demographics	Education and employment
1	42 (19%)	Young women and men Single Without children Born in Switzerland	Employed and students Upper secondary education
2	29 (13%)	Middle-aged men Married With children Born in Switzerland and abroad	Employed Upper secondary education
3	79 (36%)	Middle-aged women Married With children Born in Switzerland	Employed Upper secondary education
4	71 (32%)	Middle-aged women Married and divorced With children Born abroad	Unemployed Mandatory education

### Surgery determinants

After an early dropout or a follow-up of at least 2 years, 99 (45%) patients did not undergo bariatric surgery. The standard multidisciplinary preoperative program lasted 12.4 months on average (standard deviation: 4.9; range: 3.0–29.4).

Psychological and dietary preoperative evaluations were highly associated with the operation status, the more favorable the evaluation, the more likely to get operated on (*p* < 0.001, Table 3). Indeed, unfavorable psychological evaluation concerned 65.5% of the non-operated patients, but only 3.6% of the operated ones. The same pattern was observed with dietary evaluations.

**Table 3:** Operation status according to socio-demographic and follow-up variables

	Operated on *N* = 122	Proportion	Non-operated on * N* = 99	Proportion	Chi^2^/Fisher’s exact test ^*^
	*N*	%	*N*	%	*P* value
Age (years)					
17–24	4/122	3.3	12/99	12.1	0.152
25–34	27/122	22.1	21/99	21.2	
35–44	34/122	27.9	23/99	23.2	
45–54	40/122	32.8	28/99	28.3	
55 +	17/122	13.9	15/99	15.2	
Gender					
Female	105/122	86.1	74/98	74.7	0.039
Male	17/122	13.9	25/98	25.3	
Marital status					
Single	32/122	26.2	30/98	30.3	0.701
Married	61/122	50.0	43/98	43.4	
Divorced/separated	25/122	20.5	24/98	24.2	
Widowed	4/122	3.3	2/98	2.0	
Parenthood					
Yes	92/117	78.6	57/73	78.1	0.999
No	25/117	21.4	16/73	21.9	
Country of birth					
Switzerland	63/113	55.8	29/56	51.8	0.651
Portugal	25/113	22.1	11/56	19.6	
Other (21 countries)	25/113	22.1	16/56	28.6	
Education					
Mandatory school	36/110	32.7	9/33	27.3	0.595
Upper secondary level	64/110	58.2	19/33	57.6	
Tertiary level	10/110	9.1	5/33	15.2	
Employment status					
Employed	68/122	55.7	49/99	49.5	0.174
Stay at home	20/122	16.4	11/99	11.1	
Disability insurance	10/122	8.2	14/99	14.1	
Social aid	7/122	5.7	12/99	12.1	
Employment insurance	11/122	9.0	5/99	5.1	
At school	2/122	1.6	5/99	5.1	
Retired	4/122	3.3	3/99	3.0	
BMI (kg/m^2^)					
30–34	2/122	1.6	5/98	5.1	
35–39	38/122	31.1	37/98	37.8	
40–44	50/122	41.0	32/98	32.7	0.152
45–49	24/122	19.7	16/98	16.3	
50 +	8/122	6.6	8/98	8.2	
Cluster					
Cluster 1	21/122	17.2	21/99	21.2	0.189
Cluster 2	13/122	10.7	16/99	16.2	
Cluster 3	51/122	41.8	28/99	28.3	
Cluster 4	37/122	30.3	34/99	34.3	
Psychological evaluation					
Favorable	20/110	18.2	0/29	0.0	0.000
Close postop follow-up	35/110	31.8	2/29	6.9	
Close preop follow-up	51/110	46.4	8/29	27.6	
Unfavorable	4/110	3.6	19/29	65.5	
Dietary evaluation					
Favorable	16/75	21.3	1/24	4.2	0.000
Close postop follow-up	26/75	34.7	5/24	20.8	
Close preop follow-up	32/75	42.7	9/24	37.5	
Unfavorable	1/75	1.3	9/24	37.5	

^*^Regular chi-square tests are performed by default. Should there be less than five observations in a category, a Fisher’s exact chi-squared test is computed instead

Gender was the only socio-demographic variable statistically related to surgery (*p* < 0.05), males being underrepresented among patients operated on. They represent 13.9% of the operated patients versus 25.3% of the non-operated ones. None of the clusters defined above were significantly associated with access to surgery (Table 3).

As a sensitivity analysis, multivariate models were also performed on a data subset without missing values. The main results (details not shown) were similar to the ones presented above, i.e. unfavorable psychological and dietary evaluations were highly associated with non-operation (*p* < 0.001), whereas demographic variables and clusters were not (*p* > 0.1).

## Discussion

In this study, a 45% rate of surgical non-completion is found in a Swiss bariatric program with a hindsight of at least 2 years. Non-operation is highly associated with unfavorable preoperative psychological and dietary evaluations, and to a lesser extent with the male gender. Interestingly and somehow counterintuitively, socio-demographic characteristics are not related to the operation status.

Similar or slightly higher attrition rates are found elsewhere, such as 43% in Merrell et al*.* [20] or 50% in Sala et al*.* [21]. In comparison, Gill et al*.* [22] reported a much lower rate of 11.9% in the surgical branch of a weight loss management program. Differences in the referring process, inclusion criteria in the bariatric program, or requirements to be met prior to surgery may account for these heterogeneous results.

The highly significant association between unfavorable psychological and dietary evaluations and non-operation is in line with other studies showing that patients presenting with depression [23], binge-eating disorder [24], history of substance use or anxiety [25] are less likely to get operated on. Selecting psychologically stable patients with an appeased eating behavior is essential as it leads to better surgical outcomes in terms of weight loss and adaptation to new conditions [26]. Currently, individual consultations with the psychologist often occur at the stage of operative decision-making in the OC. Shifting this evaluation to an earlier stage in the program may help to more quickly detect people at risk for attrition and to put forward a tailored process.

Preoperative evaluations are strong determinants of bariatric surgery, but not absolute ones. In the present case, the operation is exceptionally definitively denied to patients because of these evaluations. Rather, at-risk patients are required to follow a longer preoperative and personalized preparation, as explained by Pawlow et al*.* [24]. Patients may drop out at this stage because of the delay or additional efforts required. It shows that attrition can be more complex than a simple one-sided decision and it is often the result of multiple factors and actors.

In addition, as found elsewhere [16, 27], a larger proportion of female patients commit all the way to the operation, as compared to males. An explanation may be that women suffer from greater social and professional consequences of obesity, which can drive them to more actively trying to lose weight [28]. In line with that hypothesis, Libeton et al*.* [29] found that one of the main motivations of women to undergo bariatric surgery is to better their self-image, whereas men rather aim to improve their health conditions. Consequently, to lower male attrition, health benefits from bariatric surgery could be emphasized, and additional information provided.

In the present study, age and BMI are not associated with surgery completion, as found by Sala et al. [21]. Conversely, Diamant et al. [27] identified that a BMI > 40 kg/m^2^ and age > 60 years were predictors of attrition. Such discrepancies may be due to different referring or preparation processes. Each bariatric center has its own program and comparisons are delicate to draw. Very interestingly, the analyses do not reveal any relationship between non-operation and socio-demographic characteristics (except gender) or above-defined socio-demographic clusters. It could be expected that a lower educational or social position would be a barrier, as found by Doumouras et al. [16]. However, the results show that once patients have been included in the preparation program, bariatric surgery is accessible regardless of the social situation. This result may be conditional to a setting with a dedicated multidisciplinary care and the reimbursement of bariatric surgery by a universal healthcare insurance as in the present case. Access to surgery does not seem to be associated with a specific demographic profile but rather with psychological problems and related dietary impacts.

Although unrelated to the operation status, the present study shows that beyond their weight condition, patients seeking bariatric surgery represent a very specific population, mainly made up of middle-aged women with children. In addition, an important proportion is of foreign origin, which often goes hand in hand with less favorable socio-economic situations.

As obesity is more prevalent among people with a low socio-economic status [2], it is expected to find a significant share of OC patients experiencing difficult social situations. The cluster analysis further demonstrates that these characteristics vary widely among patients. Very different life circumstances must be considered. A substantial share of patients seems to have a stable social situation, for instance, cluster 3, which is made up of employed women with upper secondary degrees. On the other hand, women included in cluster 4 combine factors of potential deprivation such as elementary education, unemployment, foreign origin, and a higher divorce rate. They represent more than 30% of the total and appear to be a particularly fragile population, which does not limit their access to surgery in the OC. In an institution proposing both medical and surgical obesity treatments, Martin [30] similarly concludes that patients suffering from obesity are a heterogeneous population in terms of socio-demographic characteristics, as also demonstrated for personality traits [31].

The four cluster classification cannot be used to detect patients at risk for surgical non-completion in the OC. However, it can be valuable as an additional tool for psychologists to help understand the patient background and offer a tailored therapeutic approach. Various measures are possible to provide patients with optimal care, including specific health insurance claims, direct collaboration with social workers, appointment times adapted to childcare, or translation of documents into their native languages. The presented clusters are based on the OC patients and are likely to vary in other bariatric centers according to regional specificities. The same methodology can be applied to find locally relevant and useful clusters. Further multicenter research is necessary to explore common cluster patterns throughout bariatric centers.

## Strength and limits

The main limitation of this study lies in the high percentage of missing values and its non-random distribution, missing values being more common among non-operated patients (Fisher’s exact test; *p* < 0.05). Data were extracted from consultation reports. However, non-operated patients may drop out before getting the entire gamut of consultations and their medical record was less complete as compared to patients following the whole program. However, when performed only in patients without missing values, the analysis of non-operation factors retrieved similar results to the ones presented here. It was decided to keep all patients to remain coherent with the retrospective study design and to allow for a more informative clustering. Another limit is the use of non-standardized dietary and psychological evaluations. Despite that, the current study is able to show the importance of these appraisals in clinical practice. One of the key strengths is the implementation of a clustering approach to identifying subgroups of patients seeking bariatric surgery.

## What is already known on this subject?

Little is known about the socio-demographic profile of persons seeking bariatric surgery, as well as its potential association to non-operation, especially in Europe. Better understanding non-operation patterns may help to prevent it and to improve cost-effectiveness.

## What this study adds?

This study is one of the only ones which examines the association of education and employment status with bariatric attrition, and to propose a socio-demographic cluster analysis to investigate the profiles of candidates. It shows that under specific conditions, bariatric surgery is accessible regardless of socio-demographic condition and that patient socio-demographic profile is very heterogenous. Additionally, the literature on bariatric surgery attrition remains scarce, and more particularly so in Europe, and this study contributes to filling this gap by giving an insight into this topic in Switzerland.

## Conclusion

This study shows that with a hindsight of at least 2 years, almost half of the patients starting a bariatric approach in the OC do not get operated on, which is related to unfavorable psychological or dietary evaluations, as well as to the male gender. It also demonstrates that a significant share of OC patients combines factors of social deprivation, which has no impact on surgery completion under conditions of multidisciplinary care and universal health insurance. Such framework conditions seem important and should be fostered to ease bariatric surgery access for fragile populations. Based on this article’s results, the OC team is currently considering the reshaping of bariatric pathways and approaches. For instance, the individual psychological assessment could be shifted to an earlier stage in the program and the cluster classification could be used as a complementary tool for professionals.

## Data Availability

Not applicable.
